# A Colorimetric Enzyme-Linked Immunosorbent Assay (ELISA) Detection Platform for a Point-of-Care Dengue Detection System on a Lab-on-Compact-Disc

**DOI:** 10.3390/s150511431

**Published:** 2015-05-18

**Authors:** Aung Thiha, Fatimah Ibrahim

**Affiliations:** 1 Department of Biomedical Engineering, Faculty of Engineering, University of Malaya, 50603 Kuala Lumpur, Malaysia; E-Mail: aungthiha.bme@gmail.com; 2 Centre For Innovation in Medical Engineering, Faculty of Engineering, University of Malaya, 50603 Kuala Lumpur, Malaysia

**Keywords:** Lab-on-Compact Disc, colorimetric sensing, Enzyme Linked Immunosorbent Assay (ELISA), point-of-care testing, lab-on-a-chip (LOC), smartphone, dengue detection, optical biosensors, immunosensors

## Abstract

The enzyme-linked Immunosorbent Assay (ELISA) is the gold standard clinical diagnostic tool for the detection and quantification of protein biomarkers. However, conventional ELISA tests have drawbacks in their requirement of time, expensive equipment and expertise for operation. Hence, for the purpose of rapid, high throughput screening and point-of-care diagnosis, researchers are miniaturizing sandwich ELISA procedures on Lab-on-a-Chip and Lab-on-Compact Disc (LOCD) platforms. This paper presents a novel integrated device to detect and interpret the ELISA test results on a LOCD platform. The system applies absorption spectrophotometry to measure the absorbance (optical density) of the sample using a monochromatic light source and optical sensor. The device performs automated analysis of the results and presents absorbance values and diagnostic test results via a graphical display or via Bluetooth to a smartphone platform which also acts as controller of the device. The efficacy of the device was evaluated by performing dengue antibody IgG ELISA on 64 hospitalized patients suspected of dengue. The results demonstrate high accuracy of the device, with 95% sensitivity and 100% specificity in detection when compared with gold standard commercial ELISA microplate readers. This sensor platform represents a significant step towards establishing ELISA as a rapid, inexpensive and automatic testing method for the purpose of point-of-care-testing (POCT) in resource-limited settings.

## 1. Introduction

Enzyme linked Immunosorbent Assay (ELISA) is a widely used clinical diagnostic tool used to detect a wide range of diseases from infectious diseases to cancer biomarkers. It is described as a precise, sensitive, versatile and quantifiable diagnostic method [1]. Although there are various rapid screening test kits for antigen/antibody detection, they have lower sensitivity and specificity compared to sandwich ELISA assays [2,3]. As an example, a conventional dengue ELISA test from Standard Diagnostics Inc. has a sensitivity of 98.8% and a specificity of 99.2%, whereas the rapid test kit from the same company has a sensitivity of 94.2% and a specificity of 96.4% according to data from the Standard Diagnostics product specifications. However, conventional ELISA tests are time-consuming, need specialized laboratory equipment and significant expertise to carry out. Hence, currently, they are unfeasible to apply in rapid testing and point-of-care diagnosis. For this reasons, researchers are trying to miniature the entire ELISA procedure on Lab-on-a-Chip (LOC) or Lab-on-Compact Disc (LOCD) platforms.

**Figure 1:**
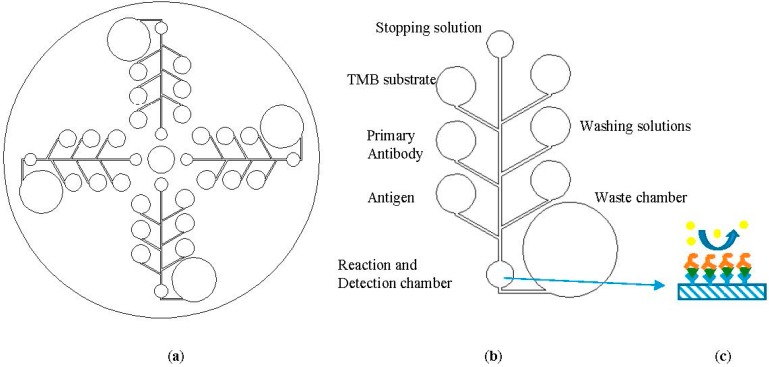
A general scheme of (**a**) a Lab-on-Compact Disc (LOCD) ELISA with four sets of assays; (**b**) a single assay showing reservoirs for various reagents; and (**c**) antigen coating on the detection chamber substrate with primary and secondary antibody attachment (adapted from [16]). The reagents are sequentially released into the reaction chamber and the end product colour change is detected by an optical sensor.

LOCD is an integrated microfluidic platform where the entire laboratory procedures can be performed on a compact disc-like structure as shown in Figure 1. In the LOCD platform, the centrifugal force of the spinning disc is used to transport the fluid from one chamber to another [4]. This LOCD structure is rotated on the spinning system with the desired velocity to manipulate fluid movement. The spinning speed (angular velocity) of the CD acts as the valve of the chambers and determines the sequence of microfluidic flow. Some of the other transport and flow control mechanisms in centrifugal microfluidic platform include the push-pull mechanism [5], pneumatic pumps [6], passive valving [7] and active valving [8]. In a CD microfluidic platform, fluid chambers and channels are embedded in a CD-like plastic structure which may be fabricated from plastics such as polymethyl methacrylate (PMMA) [5] or polydimethylsiloxane (PDMS) [9] using CNC machining.

The advantages of the LOCD platform include high throughput, low cost, rapid development and rapid testing, fully automated operation and the ability to perform multiplex testing on a single CD [4]. Moreover, a LOCD microfluidic platform can provide higher sensitivity and specificity in biosensing due to its higher surface area to volume ratio in microfluidic channels and the micro-mixing techniques that can be employed on the CD platform [10]. The LOCD platform has been tested on a wide range of applications including immunoassay [11], DNA array hybridization [9,12], cell lysis [13] and polymeric chain reaction (PCR) [8]. Our research group is focusing on developing dengue sandwich ELISA tests on test on an LOCD [14]. Dengue fever is a leading tropical disease caused by one of the four serotypes of Dengue viruses spread through *Aedes* mosquitos and infecting 100–150 millions of people annually [15]. It is one of the major health care challenges in many tropical and subtropical countries. Currently the gold standard method of dengue diagnosis is sandwich ELISA tests performed in centralized hospital laboratories. Generally, a sandwich ELISA test includes the following reaction steps: immobilization of capture antigen/antibody on a microplate, adding buffer solution, detection of antibody and several steps of washing and incubation, addition of 3,3′,5,5′-tetramethylbenzidine (TMB) and stopping solutions. These are followed by reading the absorbance (optical density (OD)) in a microplate reader. Our LOCD ELISA microfluidic assay is designed as a one-step automation of all these steps. A general scheme for a LOCD-based sandwich ELISA array is shown in Figure 1. The figure is for demonstration purposes only and it does not represent the correct schematics or flow sequence.

Although there are variety of standard commercial 96 well ELISA microplate readers for laboratory-based ELISA, there is a need for an ELISA detection system for the LOCD platform. This is because, unlike 96 wells plates, the detection chambers on a LOCD are in a circular array and they have non-uniform positions. In contrast to LOCD platforms, optical detection of ELISA has been done in lab-on-chip (LOC) platforms. Wang *et al.* [17] demonstrated ELISA optical detection with cell phone imaging. However, the chip needs to be put in a controlled lighting box. Moreover, different cellphones have different image sensor chips which will affect the image processing results. Hence, we applied a known monochromatic light emitting diode (LED) and optical sensor in our detection scheme.

In conventional ELISA, a skilled laboratory technician is needed to interpret the diagnostic results (positive or negative) as different ELISA kits have differing formulas to interpret the result from observed OD values. In order to eliminate this need in resource-poor regions, the LOCD ELISA reader ideally needs to be automated, easy-to-use and smart enough to interpret results on its own. Yang *et al.* [18] used a Charged Couple Device (CCD) camera for automated detection of standard 96 wells ELISA. The system, however, needs a computer to interpret the results. In this paper, we present a novel integrated standalone platform to read and interpret the ELISA test results on a LOCD platform. Various ELISA kits have different procedures for OD detection such as different threshold levels for distinguishing positive results from negative ones. For example, the SD dengue ELISA kit has a threshold of negative control OD value plus 2 and other kits have different values. The proposed platform can smartly interpret the results of ELISA for various ELISA kits by choosing a specific kit from an application on a smartphone, which will use the equation associated with the kit to interpret the results. The apparatus makes use of absorption spectrophotometry principle for colorimetric detection in sandwich ELISA analysis. The device also consists of a motor control system to automatically position the area of interest on the LOCD under the photodetector. The sensing system described in this work can be readily combined with control systems to achieve an integrated LOCD system that can perform the test as well as interpret the results. Since the detection system is integrated with a smartphone platform, many useful functions can be incorporated. These include smartphone-based tutorials on performing point-of-care ELISA tests, extraction of patient data, integration with healthcare information systems and various telemedicine applications.

The absorbance detection applied in the device is based on the Beer-Lambert law [19,20]. When light is transmitted through the medium, a portion of the light is absorbed. The absorbance can be calculated from the original light intensity (I_0_) and the light intensity after transmission (I) as follows:

A = −Log_10_ (I/I_0_) (1)

where,

A = absorbance of the sample,
I = transmitted light intensity,
I_0_ = original light intensity.

The greater the antigen/antibody concentration, the more light will be absorbed by the sample, resulting in a greater absorbance reading. This absorbance (OD) value is then used to interpret the diagnostic result of the ELISA.

## 2. Materials and Methods

### 2.1. System Overview

**Figure 2:**
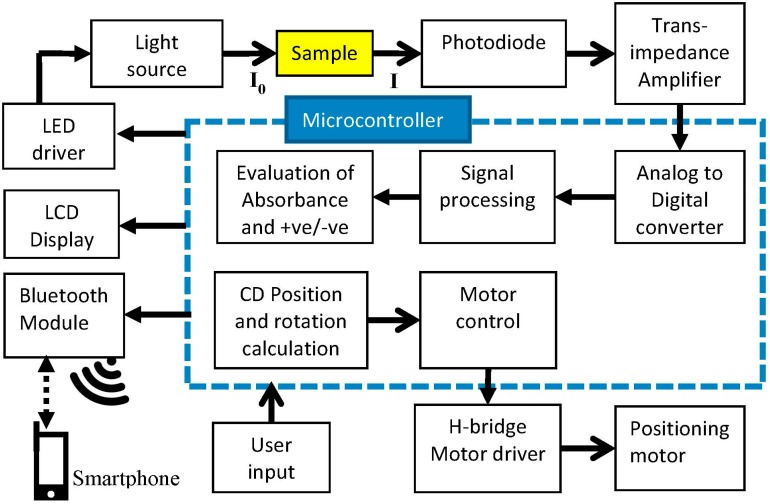
Block diagram of Colorimetric Enzyme-Linked Immunosorbent Assay (ELISA) detection platform for a Lab-on-a-Compact-Disc (LOCD) System.

The device consists of electronic circuitries of absorbance detection and motor control as well as a hardware platform where sensors and motors are placed. Block diagram of the system is described in Figure 2. The absorbance detector circuitry is comprised of a LED, a LED driver, light sensor and signal processing in the microcontroller.

A blue LED of 450 nm wavelength is used in the design since the ELISA test result using TMB and H_2_SO_4_ solution have the highest sensitivity at this wavelength. The light is passed through the sample in the microfluidic CD and the light intensity is read by the photodiode sensor. The output of the sensor is digitized by an Analog to Digital converter (ADC). The absorbance value is calculated from Beer-Lambert law using Equation (1). In this equation, I_0_ is obtained by passing the light through the water and I is obtained by passing the light through the sample. The results are then displayed on a Liquid Crystal Display (LCD) and transmitted via Bluetooth (Bluetooth SSP module from Seeedstudio, Shenzhen, China) to a smartphone. Although we demonstrated this technology with an Android phone (Nexus 4), it can also be controlled via other platforms such as iOS and Windows since the communication protocol is implemented using a common Bluetooth stack, specifically the Bluetooth Serial Port Profile (SPP). The application on a smartphone acts as the user interface of the device, displays and obtains the data, choosing a specific equation for the ELISA kit and control of the device via commands sent to a microcontroller. The platform also includes manual keypad input and a LCD display to operate independently from a phone.

The microcontroller, an ATMega328, also controls a micro-stepping stepper motor to rotate and position the detection chamber under the sensor. The required angle per rotation of the CD is calculated on the microcontroller by dividing 360° by the number of chambers. For example, the CD with 20 chambers will have 360/20 = 18° rotation per step. The microcontroller records each light intensity reading. Then, absorbance calculation and read-out of the results are carried out after all the samples are recorded. The circuitry design comprised of user-interface circuit, motor controller circuit, LED light source and photo sensor circuitry controlled by microcontroller. Motor speed and rotation are controlled by microcontroller through an H bridge Motor Driver. The H bridge circuit provides enough current for the microcontroller to drive the motor in the desired directions.

### 2.2. Experimental Procedure

Firstly, we calibrated the detection system by evaluating the sensor response to different concentrations of the samples and a standard calibration curve was constructed. The system’s sensor response was also compared to a commercial spectrophotometer over a range of wavelengths. Although the platform is designed to detect ELISA on LOCD, in order to compare with standard ELISA readers, we conducted conventional ELISA on microtiter well plates and transferred its final output solution to our fabricated LOCD for detection. The LOCD has 20 detection chambers with a depth of 10 mm which is equivalent to the depth of microplate wells. It is composed of three plastic layers fabricated from PMMA acrylic plastic by CNC machining and they are bounded by Pressure Sensitive Adhesive (PSA) layers. The construction of the CD is detailed in Section 5 of the Supplementary Materials.

**Figure 3:**
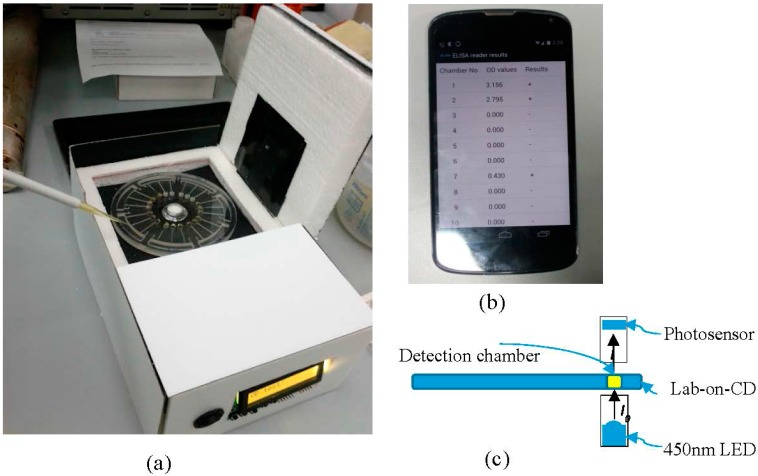
(**a**) Prototype of the LOCD ELISA reader; (**b**) Smartphone application displaying ELISA test results; (**c**) sensor operation principle in evaluating absorbance.

The laboratory standard ELISA tests were carried out for patients clinically suspected for dengue infection using the SD IgG Capture ELISA kit (Standard Diagnostic, Inc., Yongin, Korea). Three different trials on a total of 64 subjects were performed under hospital laboratory settings in the virology lab of University Malaya Medical Center by a skilled laboratory technician. We followed the test protocol as specified by the manufacturer. The procedure includes: (1) diluting 10 μL of patient samples and control samples with 990 μL of sample diluent; (2) mixing and transferring these solutions to 96 wells microplate coated with anti-human IgG; (3) incubation of the plates at 37 °C for 1 h; (4) washing of microplate with washing solution; (5) adding reacted solution of dengue antigen with anti-dengue horseradish peroxidase (HRP) conjugate from the test kit to microplates; (6) 1 h incubation at 37 °C; (7) washing of the plates; (8) addition of TMB solution which, in the presence of HRP, gives pale blue color and finally; (9) addition of stopping solution after 10 min of reaction, which turns the solution yellow. The resultant solutions were evaluated in microtiter plate reader (Model 680, Bio-Rad Laboratories Inc., Berkeley, CA, USA) for absorbance and clinical results (details of this evaluation step such as wavelength and formula are explained in the following section). Solutions were then pipetted into detection chambers in the fabricated CD to be detected by the developed system (Figure 3). The clinical performance of the device was evaluated by comparing the results with microtiter plate reader. Statistical analyses were carried out using Matlab and Analyse-it applications.

## 3. Results and Discussion

**Figure 4:**
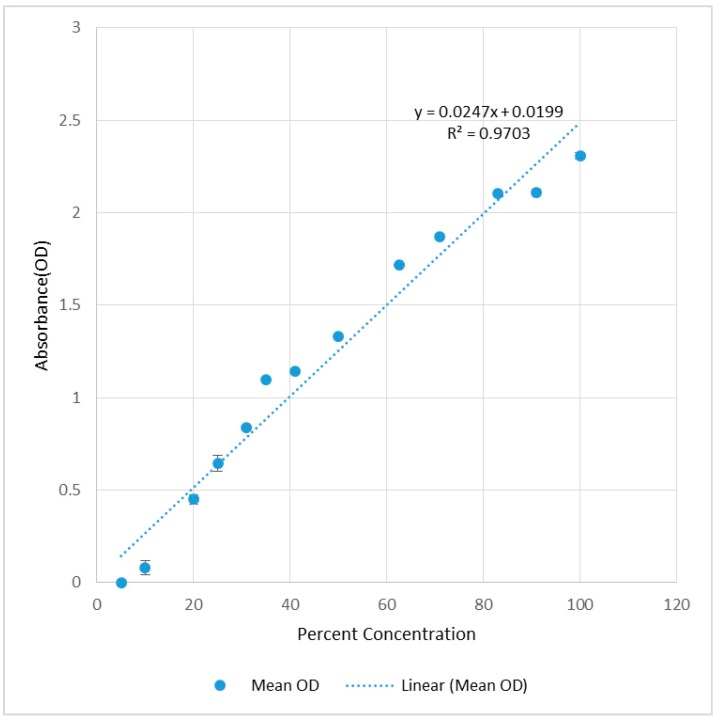
Recorded absorbance (OD) over % concentration of sample to calibrate the sensor response.

Sensor response to the concentration is constructed as shown in Figure 4 by recording the absorbance (OD) of the yellow dye solutions which are serially diluted to obtain the different concentrations (Supplementary Materials Section 1). A UV-Vis spectrum of the solution was obtained from a spectrophotometer (Uvikon 923, NorthStar Scientific, Bedfordshire, UK). The experiment was repeated three times. The mean observed OD values were compared to the theoretical OD values calculated from the percent concentration as illustrated in Figure 4. According to the Beer-Lambert law (Equation (1)), an absorbance value of 1 indicates 90% light absorption. At high solution concentrations, where absorbance will be greater than 1, the absorbance will not linearly increase with increased concentration.

We compared the absorbance detected by LOCD platform with those by conventional spectrophotometer (Uvikon 923), that can record the absorbance at a range of wavelengths from 350 nm to 700 nm. In the experiment set-up, we recorded the absorbance from 425 nm to 500 nm. The device used a blue LED which has the wavelength of 445 nm to 455 nm. Yellow dye solution was dissolved and diluted serially to get five samples. The samples were tested by both devices and the results were recorded. The absorbance spectrum from the spectrophotometer was observed and the average of absorbance values in the range of 445 nm to 455 nm was used to compare with our device. The results are presented in Table 1.

**Table 1:** Comparison of absorbance results from LOCD ELISA detection platform with absorbance values from spectrophotometer.

Mean OD of LOCD Reader at 450 nm	Mean OD of Spectrometer at 445 nm–455 nm	Error	Tolerance (%)
3.447	3.586	−0.14	−3.9
2.634	2.956	−0.323	−10.9
2.367	2.636	−0.269	−10.2
2.11	2.236	−0.126	−5.6
1.738	1.705	0.033	1.9

The absorbance reading of the developed LOCD absorbance reader device was then compared to a commercial microtiter plate reader. The absorbance (OD) of ELISA test results were first read by the standard microtiter plate reader at 450 nm with the reference wavelength at 655 nm. The samples are then transferred to the microfluidic LOCD with 20 chambers. Positive and negative results from microplate reader was evaluated by the formula given in the SD ELISA kit which is addition of 0.3 to the OD value of a negative control. The absorbance (OD) results of the samples loaded into LOCD were then read by the developed platform. The device is programmed to evaluate positive and negative results using the formula given in the ELISA kit.

To validate the absorbance reading obtained by the developed detection system, the correlation graph between the microplate reader results and our device readings is plotted and the correlation coefficient is calculated from the regression line. The regression line is found to be *y* = 1.27*x* − 0.20 with a correlation coefficient of 0.985 at 95% confidence intervals (CI). The data and graph can be found in Supplementary Materials Section 3.

The microplate reader results and subsequent evaluation indicate that out of 64 samples, 21 samples are positive to dengue while 43 samples are negative (prevalence of 0.328). Taking the microplate reader as standard, the performance of the device was evaluated showing clinical sensitivity of 95.2% and specificity of 100% at 95% confidence interval (95% CIs) as shown in Table 2.

To evaluate the predictive power of the developed device receiver operating characteristic (ROC) curve was plotted from the results and Area under ROC curve (AUROC) is calculated (See Supplementary Materials Figure S3). The AUROC of the device is found to be 1 at 95%CI, indicating the device has high prediction power in diagnosis.

**Table 2:** LOCD ELISA detection platform clinical sensitivity and specificity (n = 64). (See Supplementary Materials for details).

LOCD Reader	Outcomes	95% CI
Sensitivity—TP proportion	0.952	0.762 to 0.999
Specificity—TN proportion	1	0.918 to 1.000
FP proportion	0	0 to 0.082
FN proportion	0.048	0.001 to 0.238
Likelihood ratio (+)	+∞	
Likelihood ratio (−)	0.05	

(TP = true positive, TN = true negative, FP = false positive, FN = false negative).

A portable point-of-care sandwich ELISA test will enable highly sensitive and specific ELISA tests in rural areas and resource-limited areas. Being integrated with a smartphone, our platform can perform automated analysis and interpretation of various ELISA tests although we only have demonstrated the platform on dengue ELISA in the current paper. Moreover, it has potential to be incorporated into clinical information management systems through the internet or a mobile network. Here, we discuss the quantitative and qualitative performance of the platform.

Comparing the results to a microplate reader, a constant bias of −0.02 and proportional bias of 1.27 was observed, indicating there is a slight sensitivity drift and, hence, different quantification levels in the results. This is probably due to the single wavelength measurement design used compared to the dual wavelength measurement used in microplate readers. A correlation coefficient of 0.985 was observed.

The diagnostic outcome determined by the device has the sensitivity of 0.95 and specificity of 1.00 at 95% CI in 64 samples. With the area under Receiver Operating Characteristic (AUROC) of 1, the device shows high sensitivity, specificity and high diagnostic prediction.

Repeatability of the test results is determined from repeated the readings of the same series of samples used in the standard calibration curve over three times and regression lines’ R^2^ values of 0.98, 0.97 and 0.97 were observed. Hence, the results are comparable over different trials.

The absorbance detection range of the device is found to be lower bounded at around an optical density of about 0.3. The device gives an output of zero to any reading below that level. The upper bound range has not been specifically identified. However in the experiments we observed absorbance readings up to 3.5. The lower bounded limitation can be the result of either the single wavelength measurement or photodiode’s responsivity.

Resolution of the device is determined from the digitization capability of the analog to digital converter (ADC) of the microcontroller which has 10 bit resolution. The sensor output at maximum blue LED luminance was found to be 853 digital results out of 1024 for 10 bit. Hence the possible resolution of absorbance (OD) is found to be 0.0005 (minimum absorbance change A = log (853/852)). The device is designed to give a read out precision of three digits which is widely used in commercial microplate readers.

## 4. Conclusions

In summary, we have developed a fully automated and smart point-of-care Colorimetric Enzyme-Linked Immunosorbent Assay (ELISA) detection system for a Lab-on-a-Compact-Disc platform. This integrated platform can read and interpret the ELISA results with minimal input from the user via a smartphone application. The ELISA reader developed is evaluated to have clinical sensitivity of 95.2% and specificity of 100%. Since the device is portable, low cost and can be easily manufactured, it is well suited for point-of-care diagnosis, especially in resource-poor settings. The current prototype needs further improvement in quantitative measurement results as well as improvement in its minimum detection limit. These limitations could be eliminated in future by the use of high end photodiode chips and multiple wavelength detection. However, for the qualitative results, used in actual clinical diagnosis, our device has been shown to be very accurate and precise. Although we have demonstrated the system for dengue ELISA detection, it can be easily adapted to suit various colorimetric ELISA tests. A future step would be integration of the current detection system with a LOCD ELISA spinning system so that ELISA procedure as well as detection can be performed in one single device.

## Supplementary Files

Supplementary File 1
