# Supplementary material for: A Colorimetric Enzyme-Linked Immunosorbent Assay (ELISA) Detection Platform for a Point-of-Care Dengue Detection System on a Lab-on-Compact-Disc
Source: Sensors (Basel). 2015 May 18;15(5):11431–41. doi: 10.3390/s150511431 (PMC4481904; doi:10.3390/s150511431)
Supplement: Supplementary File 1 [file sensors-15-11431-s001.pdf]

OPEN ACCESS

sensors

ISSN 1424-8220

www.mdpi.com/journal/sensors

Supplementary Information

Colorimetric Enzyme-Linked Immunosorbent Assay (ELISA) Detection Platform for Point-of-Care Dengue Detection System on Lab-on-Compact-Disc. *Sensors* 2015, *15*, 11431-11441

Aung Thiha [1,2] and Fatimah Ibrahim [1,2,*]

[1] Department of Biomedical Engineering, Faculty of Engineering, University of Malaya, 50603 Kuala Lumpur, Malaysia; E-Mail: aungthiha.bme@gmail.com

[2] Centre For Innovation in Medical Engineering, Faculty of Engineering, University of Malaya, 50603 Kuala Lumpur, Malaysia

* Author to whom correspondence should be addressed; E-Mail: fatimah@um.edu.my; Tel.: +603-7967-6818; Fax: +603-7967-4579.

1. Sensor Response to Concentration

Table S1. Absorbance values over different concentrations.

	OD Trial 1	OD Trial 2	Mean OD	Estimated OD	Error	Accuracy (%)	Concentration	% Concentration
1	2.297	2.321	2.309	2.309	0.000	0.000	1.000	100.000
2	2.095	2.120	2.108	2.101	−0.006	−0.300	0.910	91.000
3	2.102	2.109	2.106	1.916	−0.189	−9.863	0.830	83.000
4	1.868	1.869	1.869	1.639	−0.229	−13.975	0.710	71.000
5	1.710	1.719	1.715	1.443	−0.271	−18.805	0.625	62.500
6	1.323	1.337	1.330	1.155	−0.176	−15.201	0.500	50.000
7	1.139	1.149	1.144	0.947	−0.197	−20.842	0.410	41.000
8	1.094	1.100	1.097	0.808	−0.289	−35.742	0.350	35.000
9	0.843	0.831	0.837	0.716	−0.121	−16.934	0.310	31.000
10	0.613	0.672	0.643	0.577	−0.065	−11.304	0.250	25.000
11	0.430	0.467	0.449	0.462	0.013	2.880	0.200	20.000
12	0.053	0.107	0.080	0.231	0.151	65.353	0.100	10.000
13	0.000	0.000	0.000	0.115	0.115	100.000	0.050	5.000

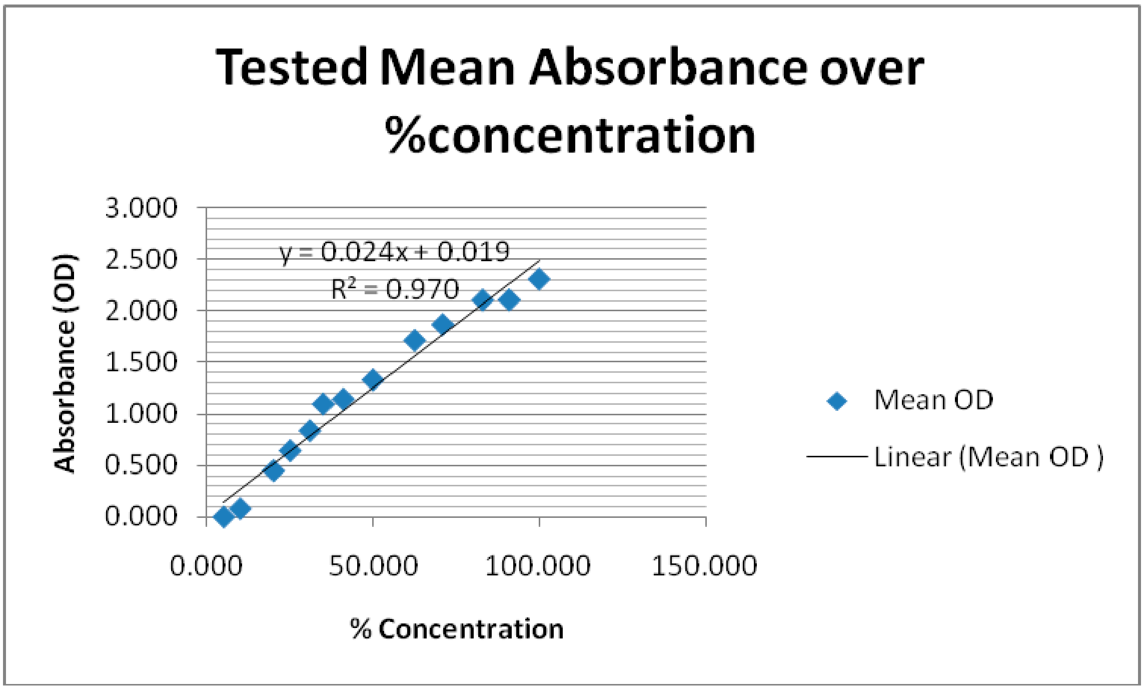

Figure S1. Recorded absorbance (OD) over per cent concentration of sample to calibrate sensor response.

2. Clinical Trial Results

Table S2. Clinical trial results.

Sample	Microplate Reader	LOCD Reader	Microplate Reader	LOCD Reader	Remark
**1**	2.397	3.156	+	+	+Control
**2**	1.999	2.795	+	+	+Control
**3**	0.049	0.000	−	−	−Control
**4**	0.052	0.000	−	−	−Control
**5**	0.056	0.000	−	−	
**6**	0.071	0.000	−	−	
**7**	0.613	0.430	+	+	
**8**	0.091	0.000	−	−	
**9**	0.062	0.000	−	−	
**10**	0.205	0.000	−	−	
**11**	0.075	0.000	−	−	
**12**	0.060	0.000	−	−	
**13**	0.066	0.000	−	−	
**14**	0.070	0.000	−	−	
**15**	1.838	2.561	+	+	
**16**	1.169	1.498	+	+	
**17**	0.088	0.000	−	−	
**18**	0.034	0.000	−	−	
**19**	0.695	0.589	+	+	
**20**	0.061	0.000	−	−	
**21**	2.987	3.665	+	+	
**22**	2.946	3.589	+	+	
**23**	0.173	0.000	−	−	
**24**	0.193	0.000	−	−	
**25**	0.219	0.000	−	−	
**26**	0.213	0.001	−	−	
**27**	0.216	0.008	−	−	
**28**	0.249	0.001	−	−	
**29**	0.358	0.001	−	−	
**30**	0.135	0.001	−	−	
**31**	0.252	0.001	−	−	
**32**	0.211	0.001	−	−	
**33**	0.406	0.001	−	−	
**34**	0.222	0.001	−	−	
**35**	0.272	0.001	−	−	
**36**	0.221	0.259	−	−	
**37**	0.360	0.240	−	−	
**38**	0.313	0.167	−	−	
**39**	0.233	0.000	−	−	
**40**	0.522	0.357	+	+	
**41**	2.601	2.774	+	+	
**42**	2.267	2.002	+	+	

Table S2. *Cont.*

**43**	0.059	0.000	−	−
**44**	0.055	0.000	−	−
**45**	0.058	0.000	−	−
**46**	0.212	0.001	−	−
**47**	0.191	0.000	−	−
**48**	0.087	0.000	−	−
**49**	0.086	0.000	−	−
**50**	1.906	2.199	+	+
**51**	0.868	0.789	+	+
**52**	2.598	3.179	+	+
**53**	0.069	0.000	−	−
**54**	0.708	0.420	+	+
**55**	0.591	0.450	+	+
**56**	0.084	0.000	−	−
**57**	0.168	0.000	−	−
**58**	0.124	0.000	−	−
**59**	1.849	2.265	+	+
**60**	0.657	0.345	+	+
**61**	0.172	0.000	−	−
**62**	0.105	0.000	−	−
**63**	0.541	0.360	+	+
**64**	0.520	0.195	+	−
**65**	0.213	0.149	−	−
**66**	0.582	0.430	+	+
**67**	0.193	0.000	−	−
**68**	0.612	0.554	+	+

3. Statistical Analysis Results in Analyze-It Software

Table S3. Regression line characteristics.

	Bias	95% CI	SE	*p*
**Constant**	−0.2	−0.25 to −0.15	0.027	<0.0001
**Proportional**	1.27	1.21 to 1.32	0.027	<0.0001
**Range**	0.034 to 0.9870			
**r**	0.985			
**Sy|x**	0.1765			

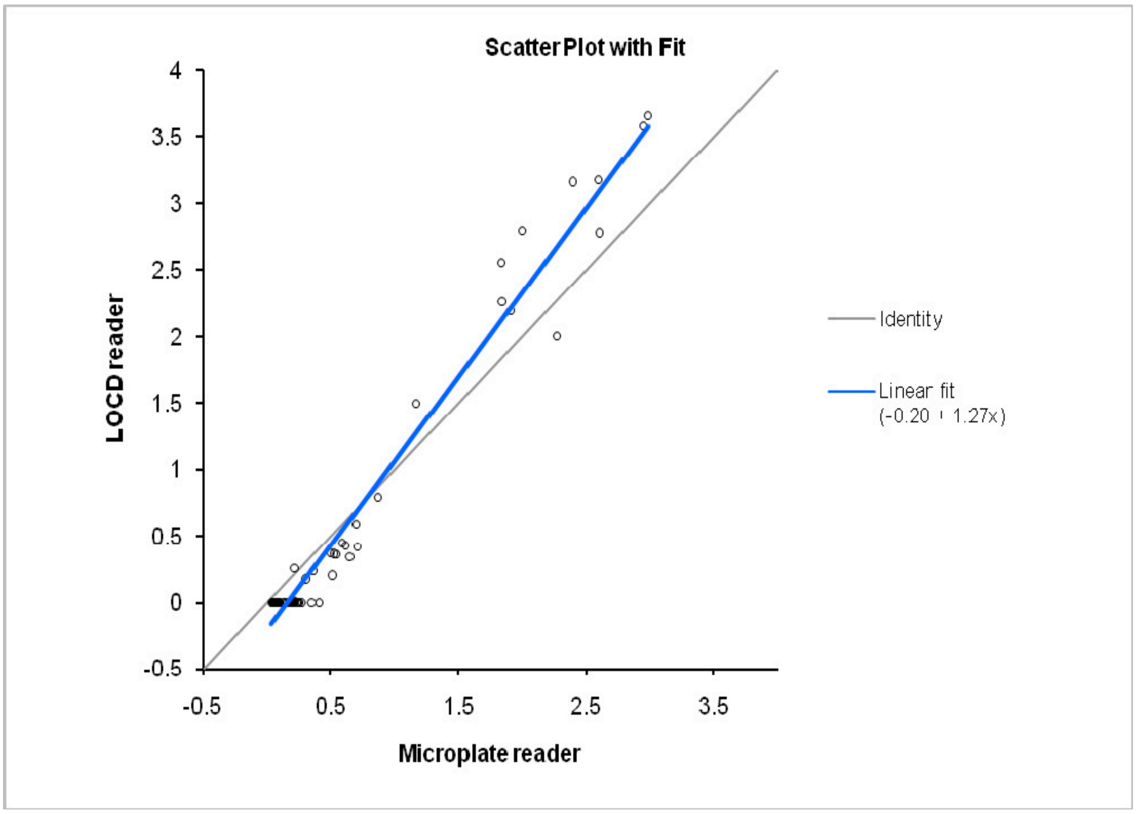

Figure S2. Correlation and linearity of the result.

Table S4. Results of LOCD reader device compared to a microplate reader.

		**Microplate Reader**		
		**Dengue +**	**Dengue −**	**Total**
**LOCD reader**	Dengue +	20	0	20
	Dengue −	1	43	44
	Total	21	43	64

Table S5. LOCD reader qualitative statistical analysis outcome.

LOCD Reader	Outcomes	95% CI
Sensitivity—TP proportion	0.952	0.762 to 0.999
Specificity—TN proportion	1	0.918 to 1.000
FP proportion	0	0 to 0.082
FN proportion	0.048	0.001 to 0.238
Likelihood ratio (+)	+∞	
Likelihood ratio (−)	0.05	

(TP = true positive, TN = true negative, FP = false positive, FN = false negative).

Table S6. Receiver Operating Characteristic (ROC) analyses of the result.

Test	Area	95% CI	SE	Dengue = +	
Microplate reader	1	1 to 1.00	0.002	have higher values	
LOCD reader	1	1 to 1.00	0	have higher values	
**Contrast**	**Difference**	**95% CI**	**SE**	**Z**	***p***
Microplate reader v LOCD reader	0	0 to 0.00	0.002	−0.71	0.4795

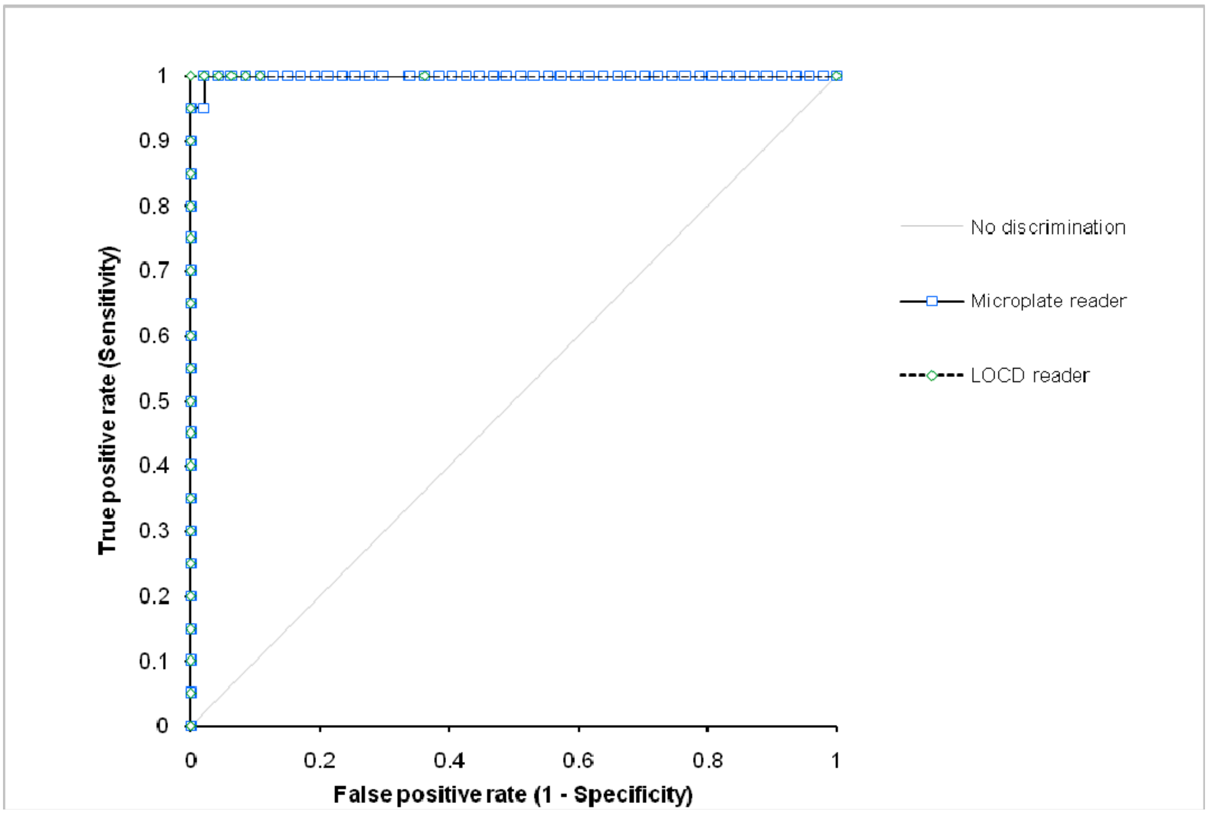

Figure S3. ROC curves of LOCD and Microplate reader results from sample of n = 64.

4. Flowchart to Evaluate Clinical Diagnostic Results

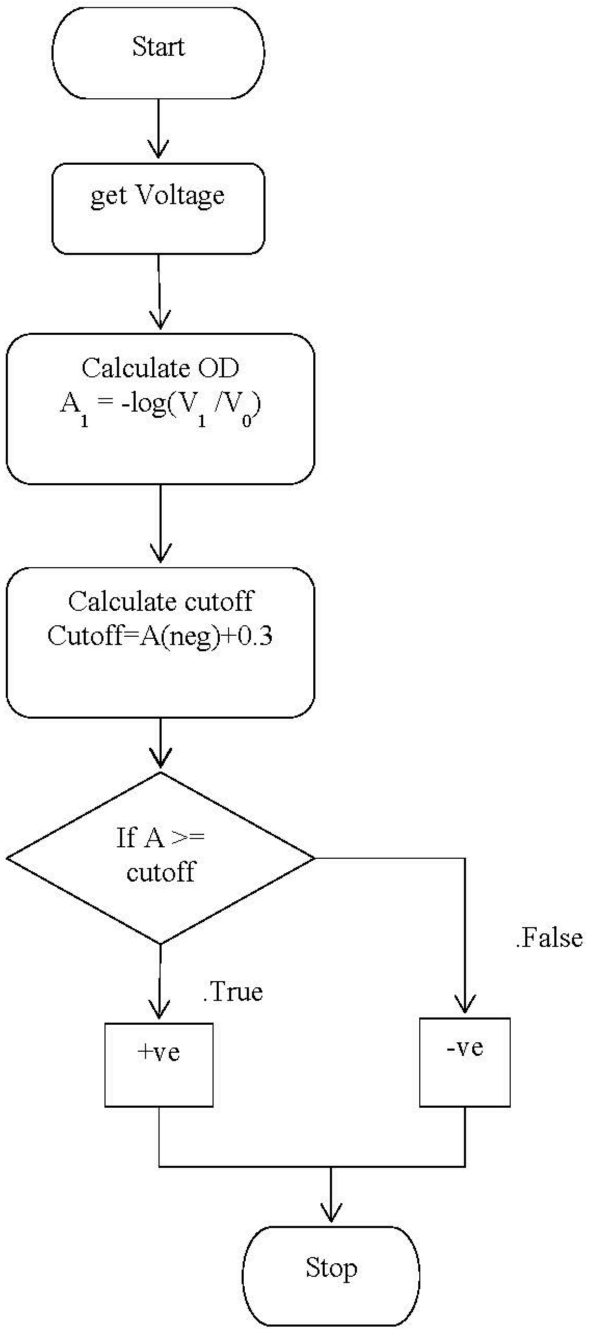

Figure S4. Flowchart to evaluate clinical diagnostic results.

5. Design and Construction of the CD for Evaluation of the Constructed Sensor

CD is constructed from three layers of PMMA bonded by two Pressure Sensitive Adhesive (PSA) layers. The CD is designed in computer aided drawing and cut by a CNC machine. The layers are bonded by applying a pressure activated PSA. The device has 20 chambers which have the same dimensions as the wells in a microtiter plate. The CD has a diameter of 130 mm and overall height of 16 mm. This CD is specifically designed for evaluation of the sensor.

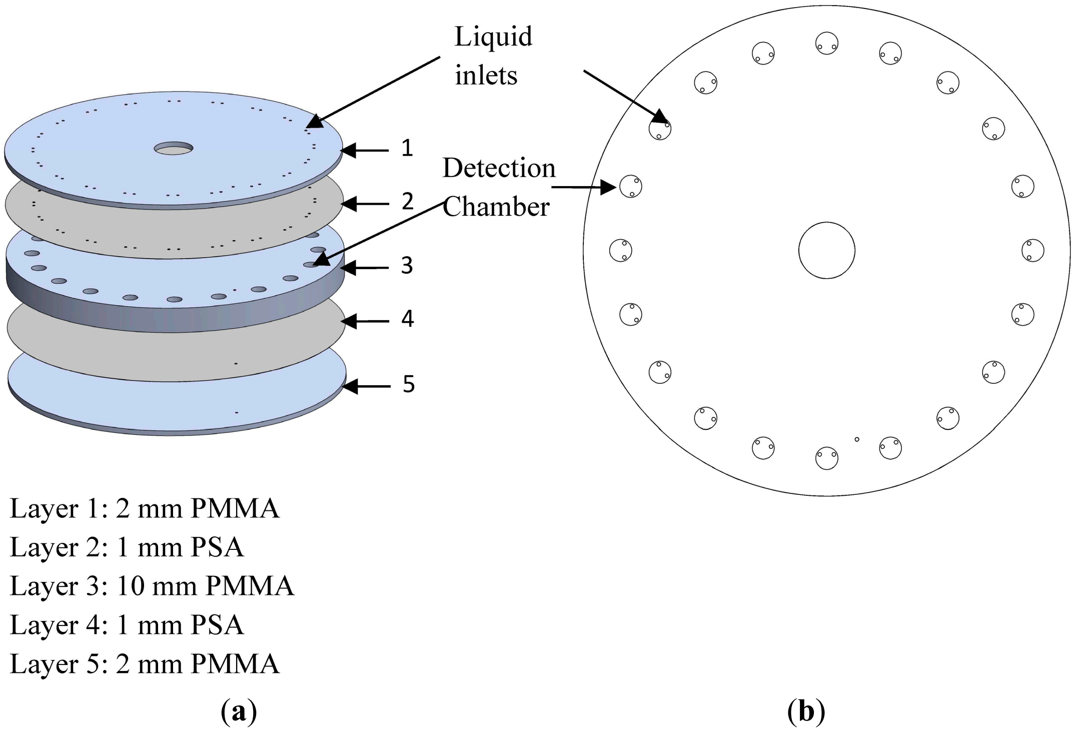

Layer 1: 2 mm PMMA
Layer 2: 1 mm PSA
Layer 3: 10 mm PMMA
Layer 4: 1 mm PSA
Layer 5: 2 mm PMMA

(**a**) (**b**)

Figure S5. (**a**) Exploded view of Lab-on Compact Disc (LOCD) for evaluation of the sensor; (**b**) 2D top view of CD.

6. Circuit Diagram of the Detection System for ELISA on LOCD

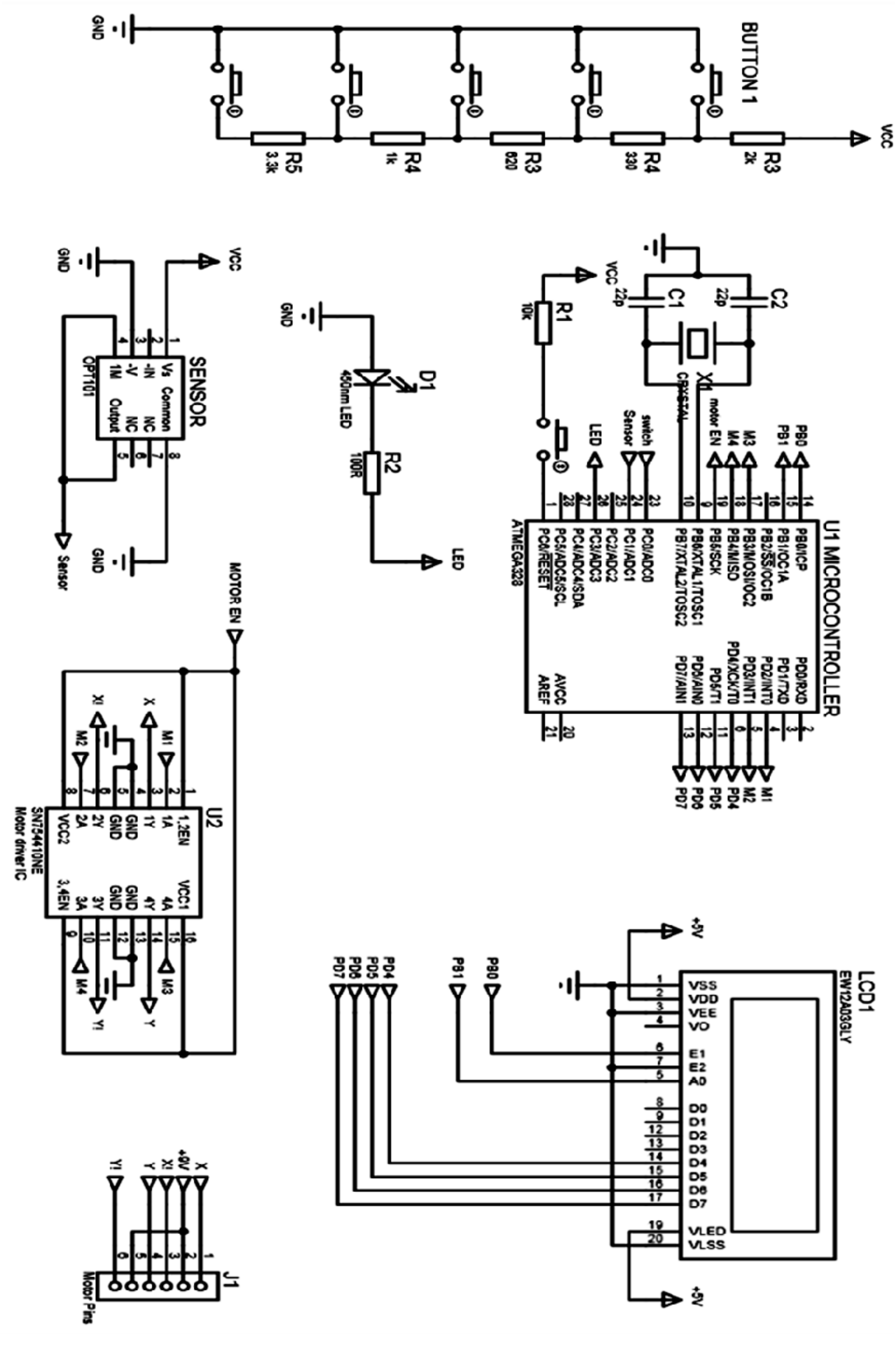

Figure S6. Circuit diagram of the detection system for ELISA on LOCD.

© 2015 by the authors; licensee MDPI, Basel, Switzerland. This article is an open access article distributed under the terms and conditions of the Creative Commons Attribution license (http://creativecommons.org/licenses/by/4.0/).
